# Evolution of 4π-Periodic Supercurrent in the Presence of an In-Plane Magnetic Field

**DOI:** 10.1021/acsnano.2c10880

**Published:** 2023-02-17

**Authors:** Bassel Heiba Elfeky, Joseph J. Cuozzo, Neda Lotfizadeh, William F. Schiela, Seyed M. Farzaneh, William M. Strickland, Dylan Langone, Enrico Rossi, Javad Shabani

**Affiliations:** † Center for Quantum Information Physics, Department of Physics, New York University, New York, New York 10003, United States; ‡ Department of Physics, William & Mary, Williamsburg, Virginia 23187, United States

**Keywords:** Josephson junction, missing Shapiro steps, spin−orbit coupling, Landau−Zener transitions, topological superconductivity

## Abstract

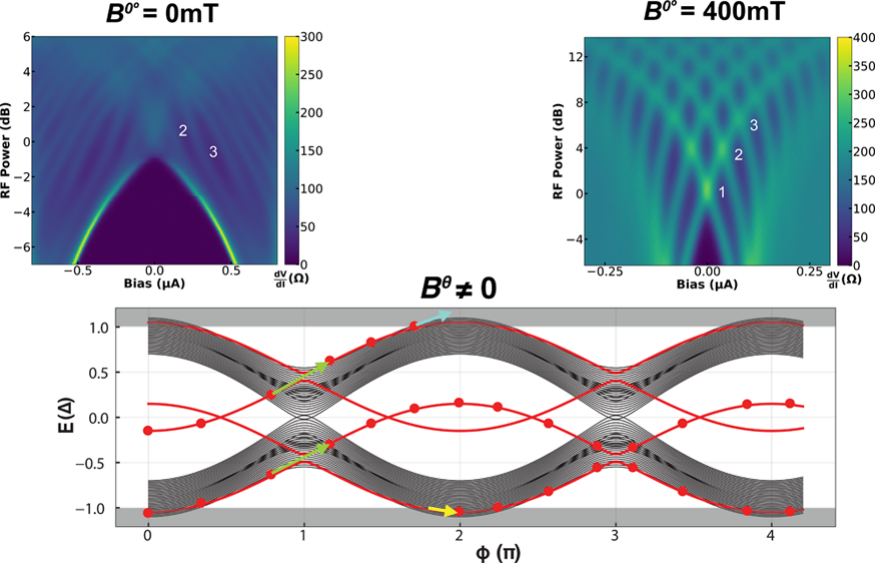

In the presence of a 4π-periodic contribution to the current phase relation, for example in topological Josephson junctions, odd Shapiro steps are expected to be missing. While missing odd Shapiro steps have been observed in several material systems and interpreted in the context of topological superconductivity, they have also been observed in topologically trivial junctions. Here, we study the evolution of such trivial missing odd Shapiro steps in Al–InAs junctions in the presence of an in-plane magnetic field *B*^θ^. We find that the odd steps reappear at a crossover *B*^θ^ value, exhibiting an in-plane field angle anisotropy that depends on spin–orbit coupling effects. We interpret this behavior by theoretically analyzing the Andreev bound state spectrum and the transitions induced by the nonadiabatic dynamics of the junction and attribute the observed anisotropy to mode-to-mode coupling. Our results highlight the complex phenomenology of missing Shapiro steps and the underlying current phase relations in planar Josephson junctions designed to realize Majorana states.

## Introduction

Josephson junctions (JJs) fabricated on semiconductor structures with epitaxially grown superconductors have recently attracted attention due to their propitious characteristics^1−9^ and applications in quantum computing.^10−18^ In the presence of a Zeeman field^19−21^ or a phase bias^22−24^ and a strong spin–orbit coupling (SOC) interaction, such high-quality JJs have shown signatures of topological superconductivity,^21−24^ which can host Majorana zero modes useful for fault-tolerant quantum computation.^25,26^ However, robust implementation and signatures of topological superconductivity remain ambiguous.^27−31^

To harness the potential of topological superconductivity, it is essential to be able to identify unambiguously the topological character of the states in a JJ. Topological JJs exhibit a fractional Josephson effect which is inaccessible with DC measurements due to relaxation processes to the ground state. Consequently, detecting the fractional Josephson effect requires measurements on time scales shorter than the relaxation time,^32−37^ time scales that are accessible using microwave excitations.^38−42^

When a microwave bias is applied to a JJ, the periodic modulation of the current bias becomes phase locked with the dynamics of the junction and results in constant voltage steps in the voltage–current characteristic known as Shapiro steps. The Andreev bound states (ABSs) of a conventional JJ in the short ballistic regime are 2π-periodic in phase ϕ, resulting in Shapiro steps at values of *nhf*/2*e*, where *f* is the frequency of the microwave drive and *n* is an integer. When the current phase relation (CPR) is 4π-periodic, as expected for a topological JJ, the fractional Josephson effect results in Shapiro steps only at *nhf*/*e*, resulting in missing odd Shapiro steps. Missing Shapiro steps have been observed in different material systems and are usually attributed to the presence of a topological state.^38−40,42−44^ In practice, even for a topological JJ, a 4π-periodic component CPR coexists with a 2π-periodic component, in which case the absence of odd Shapiro steps depends on the details of the junction and the frequency and power of the microwave radiation.^45−47^

Recent work^48^ has experimentally shown that topologically trivial JJs can also exhibit missing odd Shapiro steps as predicted previously by other theoretical works.^44,45,49−51^ This can happen when ABSs with a large probability of undergoing a Landau–Zener transition (LZT) at ϕ ∼ π and a negligible probability of crossing into the continuum are present. Other mechanisms responsible for missing Shapiro steps have also been proposed involving a bias-dependent junction resistance^52^ or the presence of multiband superconducting states.^53^ Therefore, the observation of 4π-periodic supercurrent *I*_4π_ or missing Shapiro steps is a necessary signature of topological superconductivity but is not conclusive. Given that an in-plane magnetic field *B*^θ^ is one of the ingredients required to drive a JJ to a topological transition, understanding how missing Shapiro steps depend on *B*^θ^ is essential to distinguish a trivial JJ from its topological counterpart.

In this work, we present measurements on highly transparent epitaxial Al–InAs JJs in the presence of an in-plane magnetic field *B*^θ^ and SOC effects, conditions associated with inducing topological superconductivity. For *B*^θ^ = 0 mT, we observe missing odd Shapiro steps with no applied field due to the presence of a topologically trivial *I*_4π_ as observed previously.^48^ As *B*^θ^ is increased, these missing Shapiro steps eventually reappear and no topological signatures are observed up to the junction critical field *B*_c_^θ^. The reappearance of the missing steps exhibits angle anisotropies that depend on the angle-dependent *B*_c_^θ^ and carrier density associated with SOC interaction effects. Our results show the complex dependence of topologically trivial *I*_4π_ on the applied in-plane field magnitude and direction and SOC effects.

## Results and Discussion

**Figure 1:**
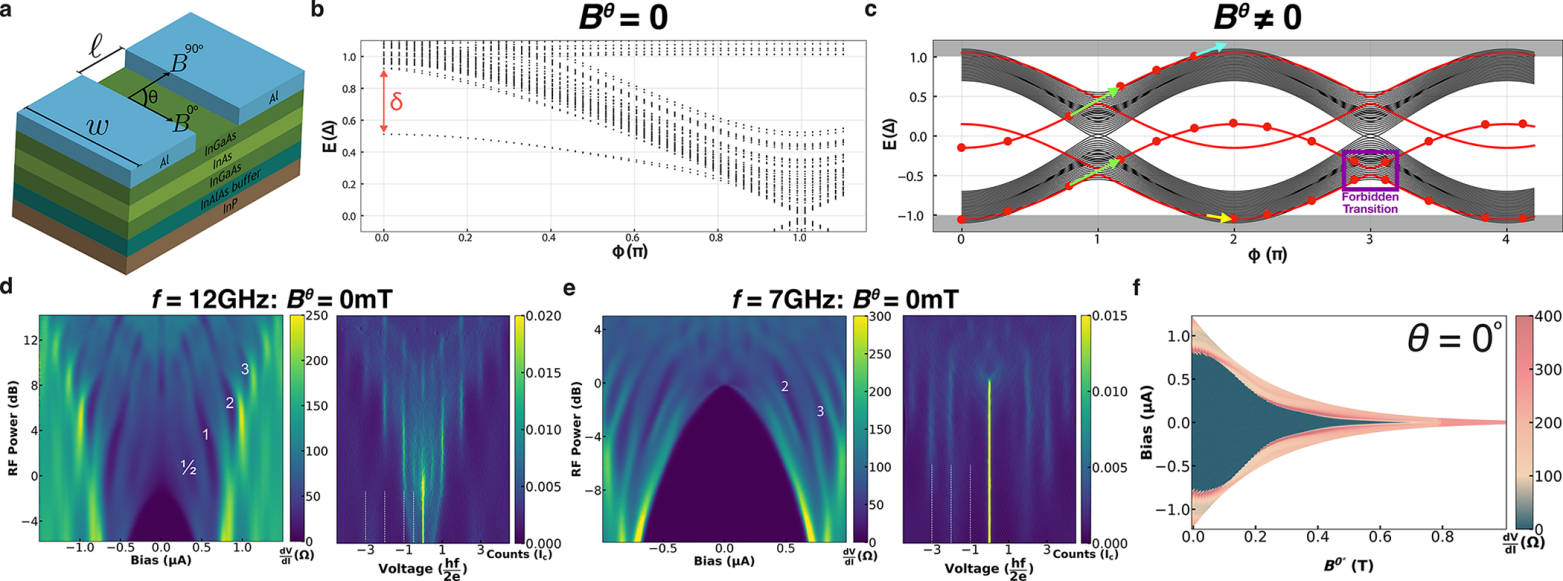
Josephson junction geometry, Andreev spectrum, and characterization. (a) Schematic drawing of the material heterostructure with a junction of width *w* and length *l* made of Al superconducting contacts and an InAs surface quantum well. The 2D axis represents the direction of an applied in-plane magnetic field, where θ is the in-plane field angle such that *B*^0°^ is the in-plane field along the junction and *B*^90°^ is the in-plane field along the current. (b) Example of calculated energy spectrum of the Andreev bound states in a wide junction with no applied magnetic field. The results obtained are for a JJ with *w* = 500 nm and *l* = 100 nm, superconducting gap Δ = 300 μeV, and carrier density *n* = 4 × 10^11^ cm^–2^. The long junction modes that contribute to *I*_4π_ are separated by δ from the quasicontinuum at *E* ∼ Δ. (c) Energy spectrum in the presence of a finite magnetic field with the spin-split long junction modes (red). The dots on the modes indicate an occupied state. Arrows indicate possible mode to mode (green) and mode to continuum (light-blue/yellow) transitions. The dark-blue arrow indicates relaxation processes that fill low-energy unoccupied states. The purple box defines a forbidden transition due to both states being occupied. (d, e) Differential resistance as a function of current bias, along with a histogram of the voltage distribution, all as a function of RF power, for frequency (d) *f* = 12 GHz and (e) *f* = 7 GHz with no applied field, *B*^θ^ = 0 mT, for JJ1. The numbers correspond to the index of the Shapiro steps. (f) Differential resistance as a function of current bias and *B*^0°^ for JJ1.

Figure 1a presents the junction heterostructure studied. An InAs near-surface quantum well is grown between two layers of In_0.81_Ga_0.19_As, which is then capped with a thin layer of epitaxial Al grown *in situ*. Two JJs, JJ1 and JJ2, are fabricated on two different wafers grown under slightly different growth conditions (see the Supporting Information). The junctions are defined using a selective wet etch of the Al and are *w* = 4 μm wide and *l* ∼ 100 nm long. Given *l* of the junctions, the calculated mean free path, to be *l*_mfp_ ≈ 150–250 nm and the superconducting coherence length ξ ≈ 530–630 nm, the junctions are expected to be in the short (*l* < ξ) ballistic (*l* < *l*_mfp_) regime.

To get insight into the dynamics of such highly transparent junctions, we first perform tight binding simulations of an Al–InAs junction using realistic parameters and calculate the energy spectrum of the ABSs shown in Figure 1b (simulation details are provided in the Supporting Information). The calculations of these wide junctions present a complex ABS spectrum with hundreds of modes. For a junction with width larger than the coherence length (*w* > ξ), modes with momentum primarily along the transverse direction behave effectively as “long junction” modes.^48^ Consequently, these modes develop a detachment gap δ from the continuum when the phase difference across the junction ϕ is zero, as indicated in Figure 1b. The number of long junction modes and their δ are sensitive to several factors (density *n*, *w*, etc.). When the junction is highly transparent, the gap at ϕ = π is sufficiently small to allow LZTs when the system is diabatically driven.^45,48,49^ The combination of a large detachment gap and a small gap at ϕ = π for these long junction modes gives rise to a 4π-periodic contribution to the CPR, causing a topologically trivial junction to have both 2π- and 4π-periodic supercurrent channels.^46,47^ In the presence of a magnetic field in the plane of the junction, the Zeeman effect splits the ABSs and eventually leads to the closing of the detachment gap of the long junction modes, as seen in Figure 1c. The 4π-periodic trajectory of long junction modes is then suppressed due to transitions to the continuum. Additionally, LZTs may occur between long junction modes and other modes with negligible detachments gaps, leading to transitions to the continuum mediated by conventional ABSs and suppressing *I*_4π_.

To experimentally investigate such trivial 4π-modes, we examine the microwave response of JJ1 in a DC current-biased setup. The measurements are carried out at *T* = 30 mK, where the junction exhibits no hysteresis, as seen in Supporting Figure S2. In Figure 1d, we present d*V*/d*I* as a function of the DC current bias and RF power at *f* = 12 GHz in addition to a histogram of the voltage distribution. For this value of *f*, we can identify all the integer Shapiro steps along with subharmonic Shapiro steps. Subharmonic Shapiro steps are expected at high frequencies due to the anharmonicity associated with the forward skewness of the CPR in highly transparent junctions.^39,54−59^ The presence of a 4π-periodic supercurrent channel, with critical current *I*_4π_, is expected to result in missing odd Shapiro steps^44,45,48−51^ when the energy of the photon irradiating the JJ, *hf*, is less than *hf*_4π_ ≈ 2*eI*_4π_*R*_*n*_.^46,47^Figure 1e shows a similar Shapiro map for *f* = 7 GHz where we see that the first odd Shapiro step is missing, indicating the presence of a finite *I*_4π_ even though the JJ is in a topologically trivial regime. For JJ1, at *B*^θ^ = 0 mT, we find *f*_4π_ ∼ 8.2 GHz corresponding to *I*_4π_ = 52.1 nA. Considering the Josephson frequency, , for JJ1, we get *f*_4π_/*f*_J_ ≅ *I*_4π_/*I*_c_ corresponding to 6.5% of the supercurrent being carried by a 4π-periodic supercurrent channel.

We next consider the dependence of the critical current *I*_c_ in JJ1 on a magnetic field, without a microwave bias, as seen in the differential resistance map in Figure 1f, where the in-plane magnetic field is applied along the junction, *B*^0°^. The critical field, , is seen to be ∼620 mT. Similar measurements performed at different θ values are presented in Supporting Figure S3. The field dependence data show no topological signatures such as a minimum in *I*_c_,^21^ indicating that the junctions are topologically trivial for all the values of *B*^θ^ up to the critical field *B*_c_^θ^.

**Figure 2:**
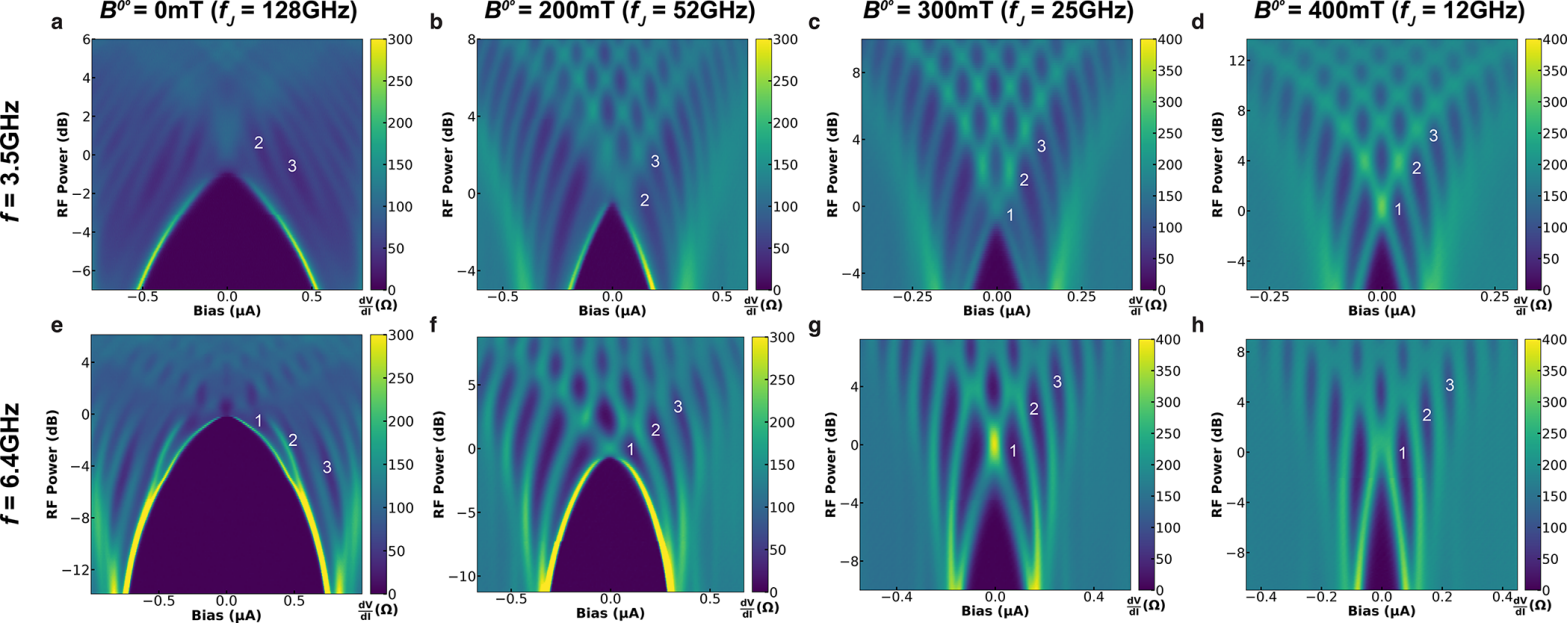
Missing Shapiro step reemergence at finite in-plane magnetic field. Differential resistance as a function of current bias and RF power at (a–d) *f* = 3.5 GHz and (e–h) *f* = 6.4 GHz for different *B*^0°^ values for JJ1.

In Figure 2, we present Shapiro maps for various magnetic field strengths applied along the junction for *f* = 3.5 GHz and *f* = 6.4 GHz. At *B*^0°^ = 0 mT, the first Shapiro step is seen to be missing for both frequencies since *f* < *f*_4π_. At *B*^0°^ = 200 mT, the first step almost completely emerges for *f* = 6.4 GHz while still being missing for *f* = 3.5 GHz. At *B*^0°^ ∼ 300 mT, the first step starts emerging for *f* = 3.5 GHz, eventually completely appearing at *B*^0°^ = 400 mT. This behavior implies a decrease of *I*_4π_ as a function of in-plane field strength, consistent with the mechanisms described in Figure 1c. We note that the data presented in Figure 2 imply that *f*_4π_ does not scale proportionally with *f*_J_. In fact, the ratio *f*_4π_/*f*_J_ generally increases as a function of in-plane field strength. This indicates that the suppression of *I*_4π_ is not simply proportional to the critical current *I*_c_, implying that the response of diabatically driven long junction modes to an in-plane field is distinct from conventional “short junction” modes that make up the rest of the spectrum in 2DEG JJs and the entire spectrum in narrow junctions, e.g., nanowire junctions.

**Figure 3:**
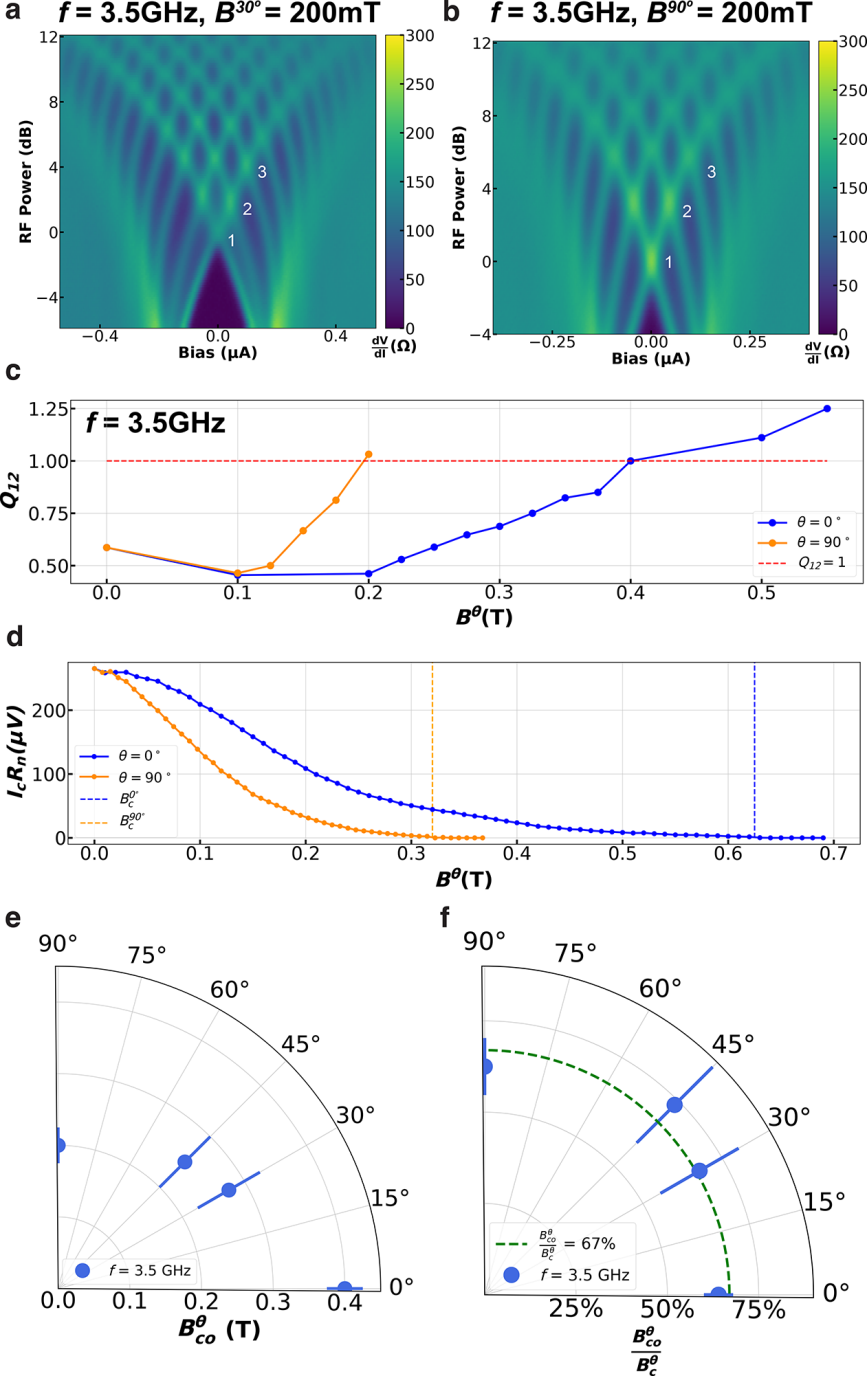
Angle dependence of reemergence of a missing Shapiro step. Shapiro maps at *B*^0°^ = 200 mT for (a) θ = 30° and (b) θ = 90°. (c) Calculated *Q*_12_ and (d) *I*_c_*R*_*n*_ as a function of in-plane magnetic field *B*^θ^ for in-plane field angles θ = 0° and 90°. (e, f) The crossover field *B*_co_^θ^, field value at which a missing Shapiro step first fully reemerges, presented in (e) units of Tesla and (f) normalized by the corresponding critical field *B*_c_^θ^, as a function of θ.

Next, we consider the *I*_4π_ dependence on the applied in-plane field direction, θ. A topologically nontrivial *I*_4π_ is expected to be sensitive^60^ to θ; on the other hand, the angle dependence of a trivial *I*_4π_ resulting from LZT is ambiguous and can depend on several contributing effects from Zeeman, orbital, and SOC interactions. Figure 3a and b show Shapiro maps with *f* = 3.5 GHz at *B*^θ^ = 200 mT for θ = 30° and θ = 90°. Unlike the θ = 0° case presented in Figure 2b, the first step appears to partially reemerge for θ = 30° and completely reemerges for θ = 90°, which indicates an angle anisotropy of *I*_4π_. To determine more precisely the threshold value of *B*^θ^ above which the first step reappears, we calculate *Q*_12_ as a function of *B*^θ^ where the ratio  represents the strength of the first step with respect to the second found by binning the voltage distribution and calculating the max step size/bin count of the first (second) step, *s*_1_ (*s*_2_). More details about the extraction of *Q*_12_ from the data are provided in the Supporting Information. We then identify the crossover field *B*_co_^θ^ for the in-plane angle θ as the value of *B*^θ^ for which *Q*_12_ ≈ 1. Figure 3c shows the evolution of *Q*_12_ with *B*^θ^ for θ = 0° and θ = 90°. In both cases, the first step is suppressed up to the crossover value *B*_co_^θ^ and is fully present for values *B*^θ^ > *B*_co_^θ^. The scaling of *Q*_12_ is seen to exhibit clear anisotropy with respect to *B*^θ^: θ = 0° shows a , whereas θ = 90° shows a *B*_co_^θ^ ≈ 200 mT.

In Figure 3e, we present a polar plot of *B*_co_^θ^ (at *f* = 3.5 GHz) as a function θ. A large variation in crossover field is observed; however, we note that the critical field *B*_c_^θ^ for θ = 0° and 90° are significantly different ( and ) as seen in Figure 3d, similar to other Al–InAs junctions.^61^ When normalized by their respective critical fields to account for the angle dependence of *B*_c_^θ^, the crossover fields become quantitatively similar and in fact match a fit of *B*_co_^θ^/*B*_c_^θ^ = 67% as seen in Figure 3f. This suggests that the anisotropy observed in *B*_co_^θ^ is likely due to the variation in critical field and implies that JJ1 has weak SOC effects.

One of the advantages of using a semiconductor-based system is the ability to have electrostatic tunability of the carrier density and SOC interaction using a gate. To study the trivial *I*_4π_ dependence on such properties, we focus on JJ2 fabricated on the same heterostructure presented in Figure 1 but equipped with a top gate. JJ2 is expected to have a stronger SOC interaction than JJ1 even at zero gate voltage (*V*_g_ = 0 V) due to the presence of a gate dielectric Al_2_O_3_ layer (see the Supporting Information) that tends to increase the carrier density and consequently SOC interaction. JJ2 is markedly hysteretic at 30 mK due to thermal effects,^62^ and so it is studied at 800 mK, where it shows no hysteresis. At *B*^θ^ = 0 mT and *V*_g_ = 0 V, JJ2 exhibits a missing first Shapiro step, as seen in Supporting Figure S8, even though at *T* = 800 mK the overall transparency is expected to be reduced. Further, Supporting Figure S4 shows that JJ2 exhibits a similar *B*_c_^θ^ anisotropy to that of JJ1. However, we note that for JJ2 *B*_c_^θ^ also depends on *V*_g_.

**Figure 4:**
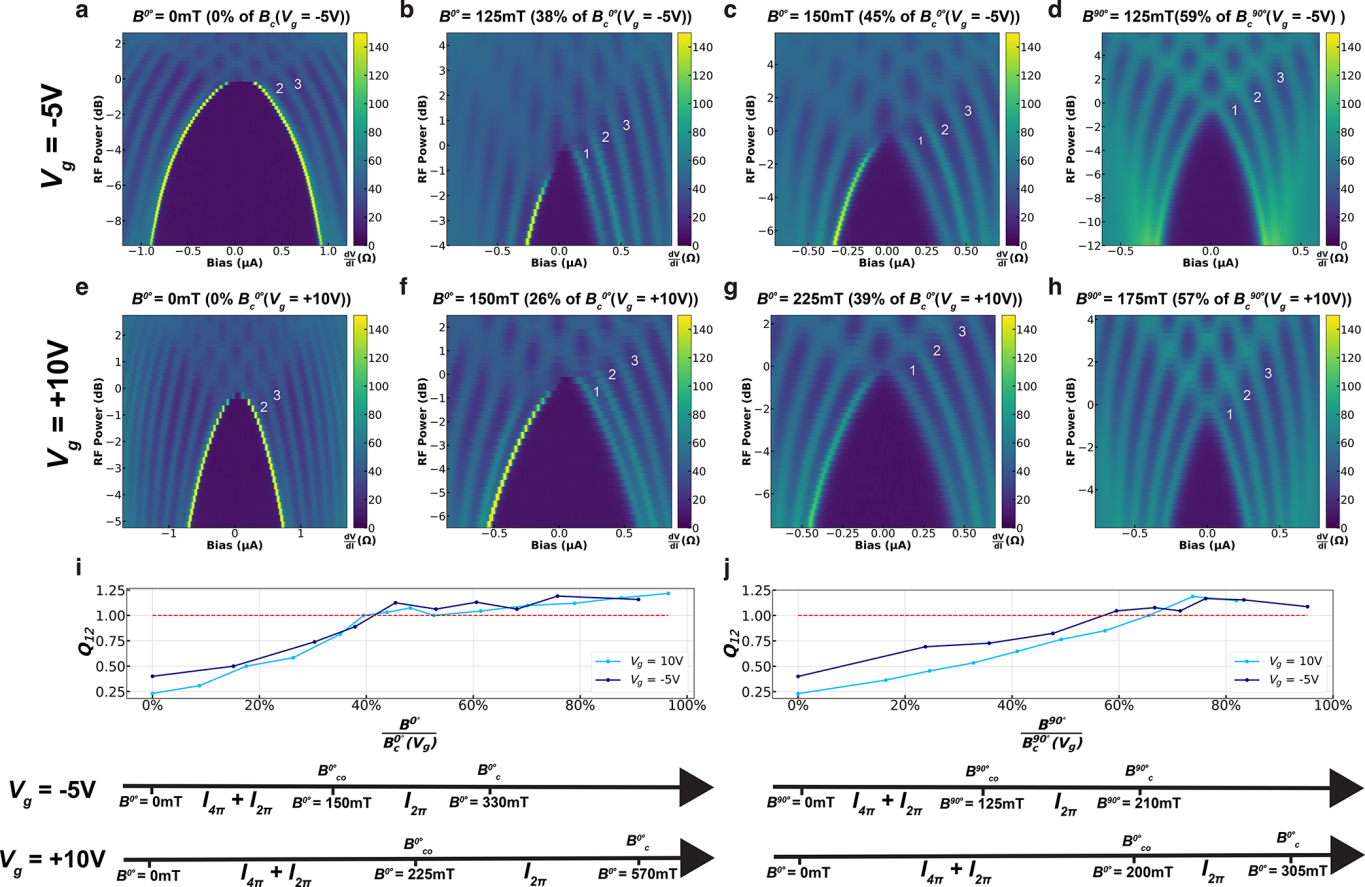
Evolution of missing Shapiro steps at an applied gate voltage for JJ2. Shapiro maps at *f* = 3.4 GHz for (a–d) *V*_g_ = −5 V and (e–h) *V*_g_ = +10 V for different *B*^0°^ and *B*^90°^ values. (i, j) *Q*_12_ as a function of *B*^θ^ (normalized by the respective critical field for each θ and *V*_g_ value) for (i) θ = 0° and (j) θ = 90°.

In Figure 4, we present measurements performed on JJ2 at *f* = 3.4 GHz for *V*_g_ = −5 V and +10 V at different *B*^0°^ and *B*^90°^ values. For θ = 0°, *V*_g_ = −5 V shows  = 125 mT, while *V*_g_ = +10 V shows . The difference between *V*_g_ = −5 V and +10 V is reconciled when considering , as seen in Figure 4i, where both *V*_g_ values exhibit a  of ∼40%. For θ = 90°, the data presented in Figure 4j show a  ratio of ∼57% and ∼65% for *V*_g_ = −5 V and +10 V, respectively. While the θ = 90° case exhibits similar  values to that reported for JJ1, the θ = 0° case shows a significant discrepancy for both *V*_g_ values. It is evident here that for JJ2 the angle anisotropy is not simply accounted for by considering *B*_c_^θ^ and that other effects play a role in the suppression of *I*_4π_, consistent with the expectation of JJ2 having stronger SOC effects in comparison to JJ1. In the following, we discuss the origin of such suppression of *I*_4π_ and the observed angle anisotropy by considering the ABS spectrum.

**Figure 5:**
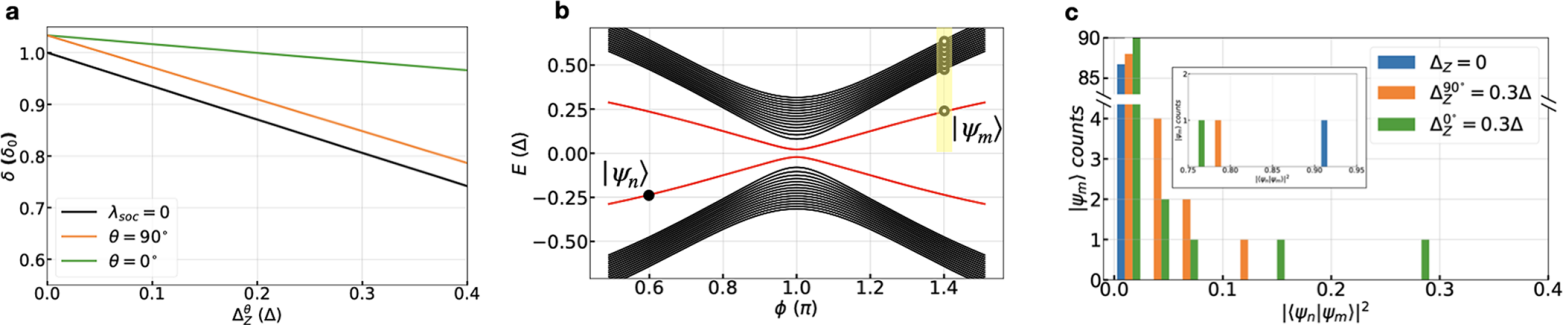
Theoretical ABS spectrum analysis in the presence of a Zeeman field. (a) Calculation of the detachment gap δ for a junction with *w* = 500 nm wide and *l* = 100 nm as a function of the Zeeman energy Δ_Z_^θ^ for λ_SO*C*_ = 0 and for θ = 0° and θ = 90° with λ_SOC_ = 7.5 meV ·nm. (b) Schematic of the Andreev bound state spectrum illustrating which states are used to calculate wave function overlaps. (c) Distribution of wave function overlaps with λ_SOC_ = 7.5 meV·nm between ϕ_i_ = 0.6π and ϕ_f_ = 1.41π for Δ_Z_^θ^ = 0 and 0.3Δ for θ = 0° and 90°. Inset: outlier overlaps where |ψ_*m*_⟩ is a long junction mode.

Following the picture presented in Figure 1c, we first consider the suppression of *I*_4π_ in terms of transitions between the long junction modes to the continuum, related mainly to the detachment gap δ. Using tight-binding simulations, we calculate the energy spectrum of the ABS spectrum in an InAs–Al junction. Figure 5a shows a linear decrease in δ as a function of the Zeeman field Δ_Z_^θ^. The decrease in δ results in a higher probability of undergoing LZTs to the continuum, suppressing the 4π-component of the CPR. In the absence of SOC effects (λ_SOC_ = 0), corresponding to the black line in Figure 5a, the suppression of δ as a function of *B*^θ^ shows no θ-dependence.

In the presence of strong SOC effects, the Fermi surface of the quantum well has an anisotropic response to an in-plane Zeeman field, creating an anisotropic suppression of δ in the ABS spectrum. For λ_SOC_ = 7.5 meV ·nm, Figure 5a illustrates that a larger Δ_Z_^θ^ in the θ = 0° (green line) direction is needed than in the θ = 90° (orange line) direction to suppress δ by the same amount. However, Figure 4i and j show . This indicates that the presence of strong SOC (as expected for JJ2) enhances the lack of correlation between the suppression of *I*_4π_ and of δ.

We thus consider the suppression of *I*_4π_ in terms of mode-to-mode coupling. Due to the large number of ABS modes in our junctions, a result of the large width *w*, we have a very dense ABS spectrum. Consequently, we have several quasi-avoided crossings between ABSs and between ABSs and the continuum. In the presence of a Zeeman field, the ABS spectrum becomes even more complex, with more quasi-avoided crossings and new protected crossings. A fully microscopic description of the JJ would require the determination of the dynamics of a multilevel Landau–Zener problem. This is a problem that is computationally prohibitive to solve.

However, to gain a qualitative understanding, we can estimate the relevant multimode couplings by calculating the wave function overlap between a long junction mode at ϕ = ϕ_i_ and all positive energy Andreev midgap states at ϕ = ϕ_f_ far from the avoided crossing at ϕ = π, as shown schematically in Figure 5b. This allows estimating the probability that an occupied ABS, when ϕ ≈ π, can either transition to an ABS with a large detachment from the continuum and therefore contribute to *I*_4π_ or transition to an ABS with a small δ and therefore contribute solely to *I*_2π_. We provide a detailed discussion of the calculations in the Supporting Information. In Figure 5c, we present a histogram of the wave function overlaps |⟨ψ_*n*_(ϕ_i_)|ψ_*m*_(ϕ_f_)⟩|^2^ between a long junction mode |ψ_*n*_⟩ and modes |ψ_*m*_⟩ for ϕ_i_ = 0.6π and ϕ_f_ = 1.41π. At Δ_Z_ = 0, we observe a distribution localized at zero except for a single outlier shown in the inset. This outlier corresponds to an overlap with another long junction mode. At finite Δ_Z_^θ^ and λ_SOC_ = 7.5 meV·nm, more states develop a nonzero overlap with the long junction mode, evident from the histogram distribution. The histogram distribution also shows that the system is more sensitive to Δ_Z_^θ^ in the θ = 0° direction than the θ = 90° direction, with the θ = 0° case exhibiting a broader distribution. These results suggest that the distribution of the overlaps between ABS states across ϕ = π, through their effect on Landau–Zener transitions, plays an important role in the anisotropy observed in Figure 4i and j for JJ2, especially where a strong SOC interaction is present.

## Conclusion

By studying the microwave response of an epitaxial Al–InAs JJ, we observe signatures of a 4π-periodic contribution to the CPR attributed to topologically trivial LZT between long junction modes. With the application of an external magnetic field, the *I*_4π_ is observed to be suppressed differently to *I*_2π_ and eventually disappears at a crossover field. In a device with weak SOC (JJ1), we observe an isotropic suppression of *I*_4π_ with an applied magnetic field when the device’s angle anisotropy in *B*_c_^θ^ is taken into account. In the gate-tunable device (JJ2) with a significantly larger SOC, an anisotropic suppression of *I*_4π_ is observed, which cannot be accounted for by the device’s *B*_c_^θ^ angle anisotropy. We attribute the anisotropy to SOC effects, which introduce a nontrivial angle θ dependence in the coupling of long junction modes to other Andreev midgap states lacking a detachment gap, suggesting multilevel LZTs. Our results indicate that such anisotropy in the in-plane magnetic field and dependence on SOC effects need to be considered when differentiating between topologically trivial and nontrivial *I*_4π_ and require other correlated signatures to make claims about topological superconductivity.

## Methods

### Fabrication Details

The devices were fabricated by electron beam lithography using a spin-coated PMMA resist. To define the mesa features of the junction, Al is removed with Transene Al etchant type-D followed by a wet etch down to the buffer layer using a III–V etchant consisting of phosphoric acid (H_3_PO_4_, 85%), hydrogen peroxide (H_2_O_2_, 30%), and deionized water in a volumetric ratio of 1:1:40. The junction gap and contacts were subsequently defined by a wet etch using Transene Al etchant type D. For JJ2, 60 nm of aluminum oxide dielectric was then deposited by atomic layer deposition to electrically isolate the gate electrodes. Next, a top gate, leads, and bonding pads consisting of 5 nm Cr and 60 nm Au were deposited via electron beam evaporation for JJ2. JJ1 does not have a dielectric layer or gates.

### Measurements Details

Our measurements are performed in an Oxford Triton dilution refrigerator fitted with a vector magnet. For JJ1, all measurements were performed at 30 mK, where there is no hysteresis observed, as seen in Figure S1b. For JJ2, all measurements were performed at 800 mK to avoid the effects of hysteresis since the junction is hysteretic at 30 mK but not 800 mK, as seen in Figure S5. Standard dc and lock-in techniques are used at low frequencies (17 Hz) with a current excitation of *I*_ac_ = 10 nA in a four-point geometry using a current-biased configuration by sweeping *I*_dc_ and the differential resistance d*V*/d*I* using an SRS860 lock-in amplifier as well as the voltage drop *V* across the junction using a Keithley DMM6500. We measure the switching or critical current at which the junction switches from the superconducting to the normal resistive state. All current bias sweeps are done from negative to positive unless specified otherwise.

## Supporting Information Available

Details on material growth, carrier density in the junctions, additional measurements, information about the theoretical analysis (PDF)
