# Supplementary material for: Evolution of 4π-Periodic Supercurrent in the Presence of an In-Plane Magnetic Field
Source: ACS Nano. 2023 Feb 17;17(5):4650–8. doi: 10.1021/acsnano.2c10880 (PMC10018771; doi:10.1021/acsnano.2c10880)
Supplement: Supplementary file 1 — nn2c10880_si_001.pdf [file nn2c10880_si_001.pdf]

Supporting Information: Evolution of 4π-periodic Supercurrent in the Presence of In-plane Magnetic Field

Bassel Heiba Elfeky,[†] Joseph J. Cuozzo,[‡] Neda Lotfizadeh,[†] William F. Schiela,[†] Seyed M. Farzaneh,[†] William M. Strickland,[†] Dylan Langone,[†] Enrico Rossi,[‡] and Javad Shabani[*,†]

†*Center for Quantum Information Physics, Department of Physics, New York University, NY 10003, USA*

‡*Department of Physics, William & Mary, Williamsburg, VA, 23187, USA*

E-mail: jshabani@nyu.edu

Wafer Structure

JJ1 and JJ2 are fabricated on wafer W1 and wafer W2, respectively, which have nominally identical structures aside from differences in the buffer layer and doping density. Structures are grown via molecular beam epitaxy. On an InP substrate, a 50 nm thick $In_{0.52}Al_{0.48}As/In_{0.53}Ga_{0.47}As$ superlattice of ten periods is grown followed by a 50 nm thick $In_{0.52}Al_{0.48}As$ layer. In both W1 and W2, an $In_xAl_{1-x}As$ step graded buffer layer with $0.52 < x < 0.81$ where the composition is graded in 50 nm steps with $\Delta x = 0.02$ is then grown at 350 - 360 °C. W2 employs a subsequent strain relaxation layer, where the composition is increased to $x = 0.85$ and decreased back to $x = 0.81$ in four 50 nm steps. W1 does not have this layer. On both W1 and W2 a 50 nm thick $In_{0.81}Al_{0.19}As$ virtual layer is then grown. The structures are then modulation-doped with Si. The Si cell temperatures are 1270 °C for W1, and 1320 °C for W2 resulting in a slightly lower measured 2D electron density for W1's 2DEG. The quantum well consists of a 4 nm bottom barrier of $In_{0.81}Ga_{0.19}As$, a 4 nm InAs layer, and a 10 nm top barrier for both W1 and W2. The wafer is subsequently cooled to -20 °C for the growth of smooth and thin Al films.

Carrier density in JJ1 and JJ2

The carrier density n of W1 and W2's 2DEG is measured in a Van-der-Pauw geometry. For W1 and W2, the density is shown to be $n = 5.97 \times 10^{11}\,\mathrm{cm}^{-2}$ and $n = 8.24 \times 10^{11}\,\mathrm{cm}^{-2}$, respectively. JJ2 has an additional dielectric Al_2O_3 layer deposited using atomic layer deposition which is not present for JJ1. The presence of the Al_2O_3 layer is expected to further increase n[1] for JJ2. The higher intrinsic n in W2 and the additional increase in n due to the Al_2O_3 layer indicates that JJ2 is expected to have significantly stronger SOC effects compared to JJ1.

Additional Measurements

Device characterization

In Fig. **S1** and Fig. **S2**, we present some additional DC measurements on JJ1 and JJ2. For JJ1, the VI characteristic in Fig. **S1**a shows agreement between the forward and backward sweeps at $T = 30\,\mathrm{mK}$ indicating the absence of hysteresis. All the measurements presented on JJ1 are performed at $T = 30\,\mathrm{mK}$. On the other hand, as seen in Fig. **S2**, JJ2 exhibits hys-

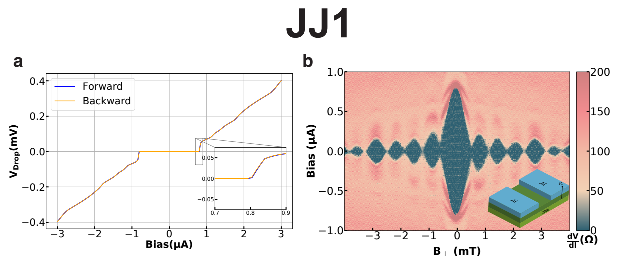

Figure **S1**: **Sample characterization of JJ1.** **a** Measured voltage drop as a function of current bias in the forward (negative to positive bias) and backward (positive to negative bias) directions at $T = 30\,\mathrm{mK}$. **b** Differential resistance as a function of current bias and out-of-plane magnetic field $B_{\perp}$. The inset illustrates the direction of the field with respect to the junction.

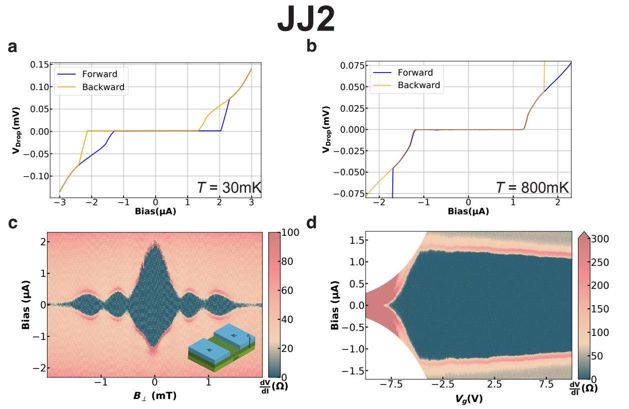

Figure **S2**: **Sample characterization of JJ2.** VI characteristic in the forward and backward directions at **a** $T = 30\,\mathrm{mK}$ where the junction is hysteretic and **b** at $T = 800\,\mathrm{mK}$ where there is no hysteresis observed. **c** Differential resistance as a function of current bias and out-of-plane magnetic field $B_{\perp}$. The inset illustrates the direction of the field with respect to the junction. **d** Differential resistance as a function of current bias and applied gate voltage V_g. For JJ2, the measurements presented in **c** and **d** as well as all the measurements presented in the manuscript are performed at $T = 800\,\mathrm{mK}$ to avoid the effects of hysteresis.

teresis at $T = 30\,\mathrm{mK}$ but not at $T = 800\,\mathrm{mK}$. Thus, for JJ2, all the measurements presented are performed at $T = 800\,\mathrm{mK}$ to avoid hysteresis effects.

As a function of out-of-plane magnetic field, the critical current exhibits a conventional Fraunhofer-like pattern as seen in the differential resistance map in Fig. **S1**b and Fig. **S2**c. Due to flux-focusing caused by the Meissner effect and London penetration,[2,3] the effective area of the junction as calculated from the periodicity of the pattern is expected to be larger than the nominal geometry.

In-plane field dependence

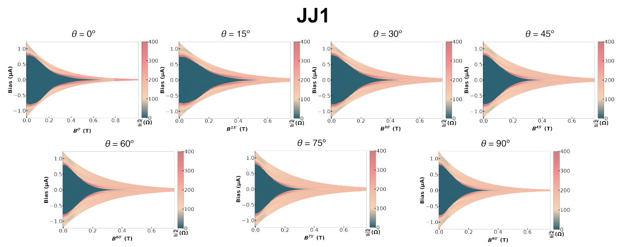

Figure **S3**: **Angle anisotropy of critical field for JJ1.** Differential resistance as a function of current bias and in-plane magnetic field B^{θ} at different values of θ. The critical field B_c^{θ}, field at which supercurrent disappears, is seen to decrease as the angle is swept from $\theta = 0°$ to $\theta = 90°$.

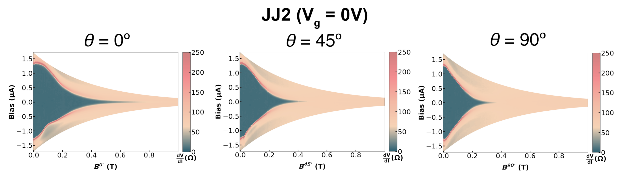

Figure **S4**: **Angle anisotropy of critical field for JJ2.** Differential resistance as a function of current bias and in-plane magnetic field B^{θ} at different values of θ.

The in-plane field data presented in Fig. **S3** and Fig. **S4** show an angle anisotropy in the critical field B_c^{θ}. This behavior has been reported in other epitaxial Al-InAs Josephson junctions and discussed in Ref.[2] A possible explanation is the induction of a flux dipole in the junction due to the bending of field lines around the contact edges.[2] This adds an additional out-of-plane field component close to the superconducting contacts affecting an electron flowing in the in-plane direction. This effect arises only when there is a component of the

in-plane field along the current, thus restricting the critical field in the $\theta \neq 0$ direction and most significantly at $\theta = 90$ as evident from the data presented.

Microwave measurements

Fig. **S5** and Fig. **S6** show a complete set of the Shapiro measurements for JJ1 for different in-plane field values and angles. In Fig. **S7**, we show how Q_{12} evolves as a function of frequency at zero and finite field. The data shows an decrease in $f_{4\pi}$ at $B^{0^\circ} = 200\,\mathrm{mT}$ while the ratio $f_{4\pi}/f_J$ doubles.

Fig. **S8** through Fig. **S13** show a complete data set of Shapiro measurements for JJ2 at $f = 3.5\,\mathrm{GHz}$ for different in-plane field values and angles at three different gate voltage values. A similar data set for $f = 2\,\mathrm{GHz}$ is shown in Fig. **S14** and Fig. **S15**.

Effect of microwave frequency range limitations

To observe missing Shapiro steps in the presence of $I_{4\pi}$, a microwave frequency below $f_{4\pi}$ is required. For both devices, we perform measurements at the lowest frequency possible to maximize the effects of $I_{4\pi}$. The RF signal is applied through an antenna in the puck that is close to the sample. Due to this coupling, the effective power radiated on the device changes from frequency to frequency. For the measurements, we choose frequencies where the entire Shapiro map is visible in our accessible power range. The lower limit of this accessible frequency range is primarily set by the weak coupling of the microwave antenna to the device at low frequencies.

The data presented in Fig. 2 of the manuscript shows a decrease in $f_{4\pi}$ with the application of an in-plane magnetic field consistent with a suppression of $I_{4\pi}$. In Fig. **S8**, we present Shapiro maps at $f = 3.4\,\mathrm{GHz}$ for JJ2 at $V_g = 0\mathrm{V}$ where the first step is seen to fully reemerge around $B^{0^\circ} \sim 175$ - $200\,\mathrm{mT}$. Measurements performed at $f = 2\,\mathrm{GHz}$ shown in Fig. **S14** also show the full reemergence of the first step around $B^{0^\circ} \sim 175$- $200\,\mathrm{mT}$. A similar agreement between $f = 3.4\,\mathrm{GHz}$ and $f = 2\,\mathrm{GHz}$ is also seen for the $\theta = 90^\circ$ case in Fig. **S9** and Fig. **S15**. This data shows that our study is not limited by the accessible frequency range of our setup. In the manuscript, we focus mainly on the $f = 3.4\,\mathrm{GHz}$ data because it is difficult to extract accurate information from the $f = 2\,\mathrm{GHz}$ data due to the size of the Shairo steps and low coupling.

Extracting Q_{12} from the experimental Data

To quantify how strong the first Shapiro step is, we calculate a ratio between the first and second Shapiro steps similar to the analysis done in Ref.[4] The strength of a Shapiro step can be quantified by binning (in fractions of $\frac{hf}{2e}$) the voltage distribution of the V-I curve and creating a histogram of bin counts as a function of power. Horizontal linecuts through the colormap of the voltage distribution (i.e., Fig. 1d and 1e in the manuscript) present the amplitude of the Shapiro steps in terms of bin counts. A high bin count corresponds to a well-defined Shapiro step, while a low count corresponds to a suppressed or missing step. As the power is increased, Shapiro steps start to become more visible while the amplitude of the supercurrent ($n = 0$) decreases and eventually vanishes. At higher power, an oscillatory pattern corresponding to Bessel functions emerges. The power range considered for this analysis is always the low power regime which is the power range below the vanishing of the supercurrent. A ratio $Q_{n,n+1} = \frac{s_n}{s_{n+1}}$, where s_n is the max step size (bin count) of step n with $n \in \mathbb{Z}$ can be calculated. A ratio of first to second step, $Q_{12} < 1$, corresponds to a suppression of the first step while the reemergence of the first step with $Q_{12} \gtrsim 1$. At low frequencies, the Shapiro steps are not, well-defined and the Shapiro steps can have s_n that is not significantly greater than the "background". Thus, even when the first step is missing, Q_{12} doesn't reach a value close to 0 at low in-plane fields.

For a specific range of B^{0°, Fig. **S5** and Fig. **S8** show a slight asymmetry around the current bias axis in terms of the position of the Shapiro steps. This asymmetry is not seen in the B^{90° case shown Fig. **S6** and Fig. **S9**. The current bias as a function of B^{0° plots pre-

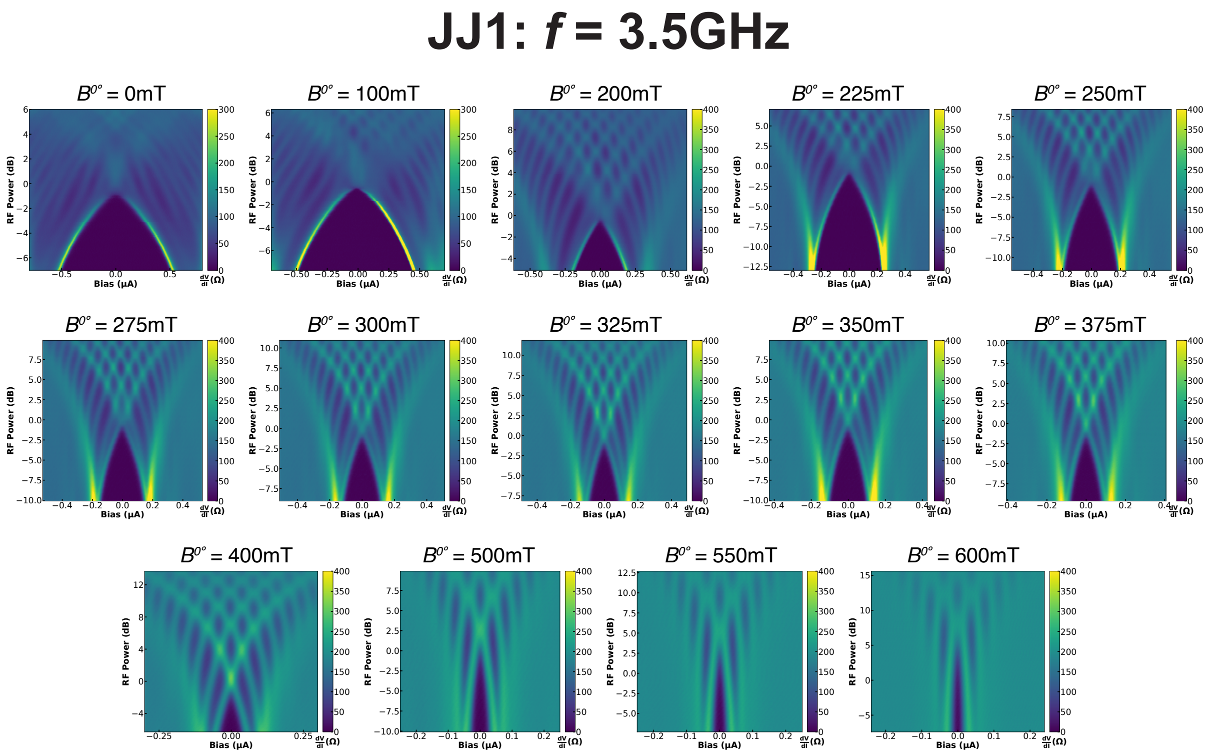

Figure **S5**: **Shapiro maps for** $f = 3.5\,\mathrm{GHz}$ **at different values of** $B^{0°}$ **for JJ1.** The first Shapiro step is seen to be missing at $= 0\,\mathrm{mT}$. The first step starts to reappear around $B^{0°} \sim 275\,\mathrm{mT}$ and gradually becomes more visible, completely reemerging (first step becoming equal to or larger in size than second step) around $B^{0°}_{co} = B^{0°} = 400\,\mathrm{mT}$. Beyond $B^{0°} = 400\,\mathrm{mT}$, all integer steps are visible.

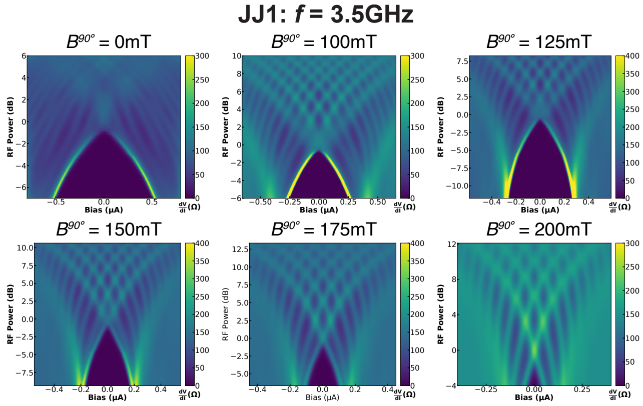

Figure **S6**: **Shapiro maps for** $f = 3.5\,\mathrm{GHz}$ **at different values of** $B^{90°}$ **for JJ1.** The first Shapiro step is missing at low $B^{90°}$ and gradually reappears starting around $B^{90°} = 150\,\mathrm{mT}$ with complete reappearance occurring around $B^{90°}_{co} = B^{90°} = 200\,\mathrm{mT}$.

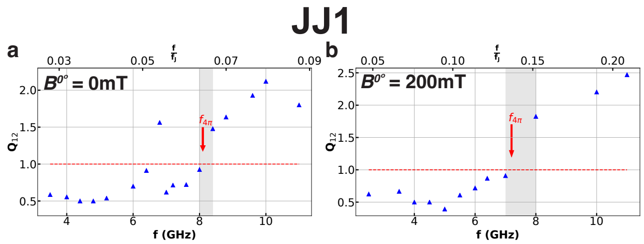

Figure **S7**: **Decrease in** $f_{4\pi}$ **at finite field.** Q_{12} as a function of drive frequency (bottom axis) and the drive frequency normalized by the Josephson frequency $\frac{f}{f_J} = \frac{hf}{2eI_c(B^\theta)R_n}$ (top axis) for **a** $B^{0°} = 0\,\mathrm{mT}$ and **b** $B^{0°} = 200\,\mathrm{mT}$ for JJ1. The shaded gray region indicates the uncertainty of the crossover frequency $f_{4\pi}$.

sented in Fig. **S3**a and Fig. **S4**a also exhibits slight asymmetries around the current bias between $B^{0°} \sim 50\,\mathrm{mT}$ and $B^{0°} \sim 200\,\mathrm{mT}$ which could contribute to the asymmetry seen in the

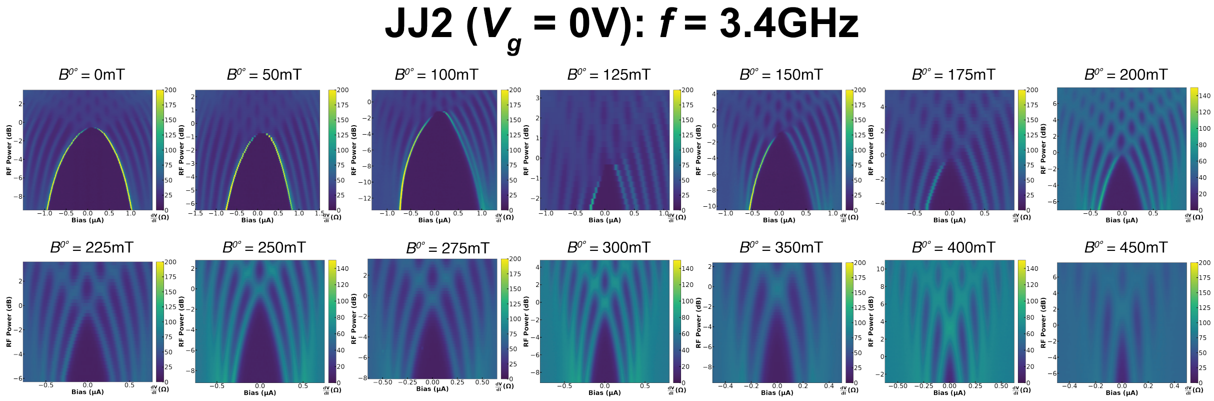

Figure **S8**: **Shapiro maps for** $f = 3.4\,\mathrm{GHz}$ **at different values of** B^{0° **for JJ2 at** $V_g = 0\mathbf{V}$**.** The first Shapiro step is seen to be missing at $0\,\mathrm{mT}$. The first step starts to reappear around $B^{0^\circ} \sim 100\,\mathrm{mT}$ and gradually becomes more visible, eventually completely reemerging with $B^{90^\circ}_{co} \sim$ 175-200 mT. Beyond $B^{0^\circ} = 200\,\mathrm{mT}$, all integer steps are visible in the Shapiro maps.

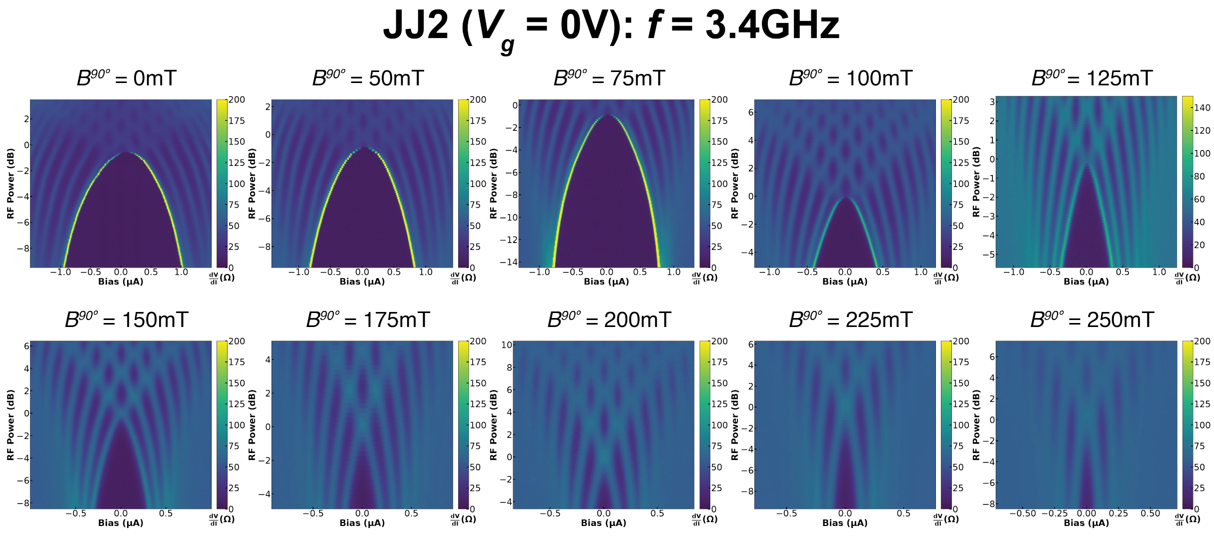

Figure **S9**: **Shapiro maps for** $f = 3.4\,\mathrm{GHz}$ **at different values of** B^{90° **for JJ2 at** $V_g = 0\mathbf{V}$**.** $B^{90^\circ}_{co}$ is seen to be $150\,\mathrm{mT}$.

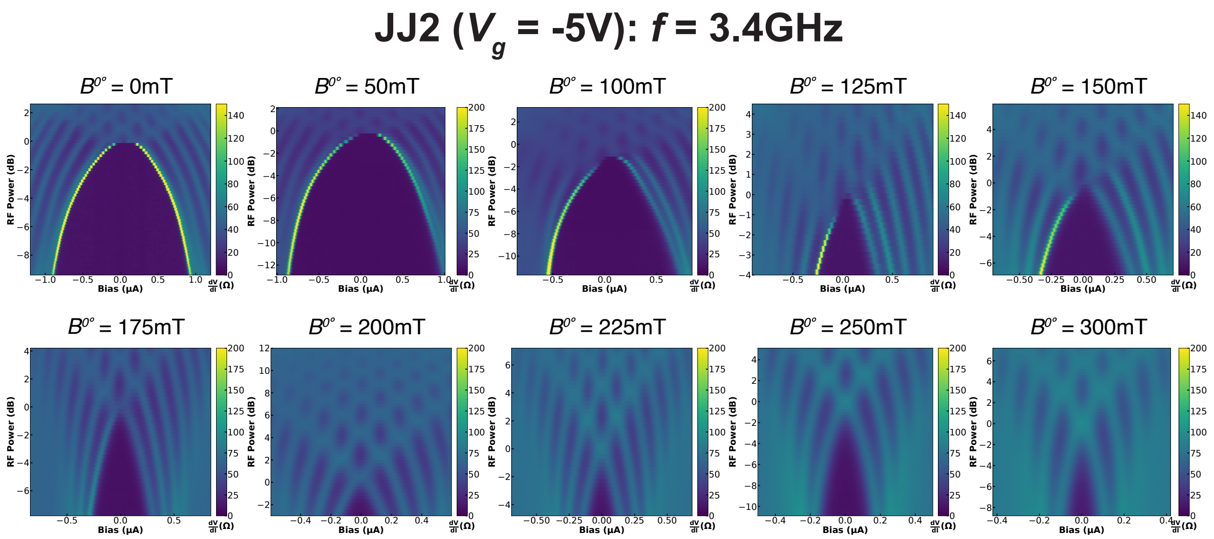

Figure **S10**: **Shapiro maps for** $f = 3.4\,\text{GHz}$ **at different values of** B^{0° **for JJ2 at** $V_g = -5$**V.** $B^{0^\circ}_{co}$ is seen to be $\sim 150\,\text{mT}$.

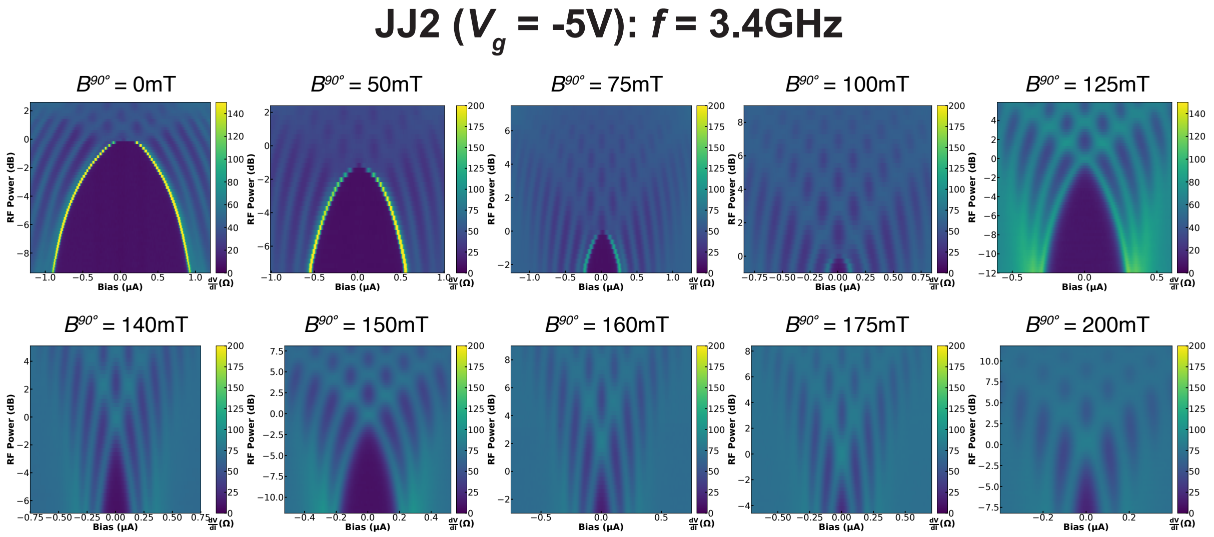

Figure **S11**: **Shapiro maps for** $f = 3.4\,\text{GHz}$ **at different values of** B^{90° **for JJ2 at** $V_g = -5$**V.** $B^{90^\circ}_{co}$ is seen to be $\sim 125\,\text{mT}$.

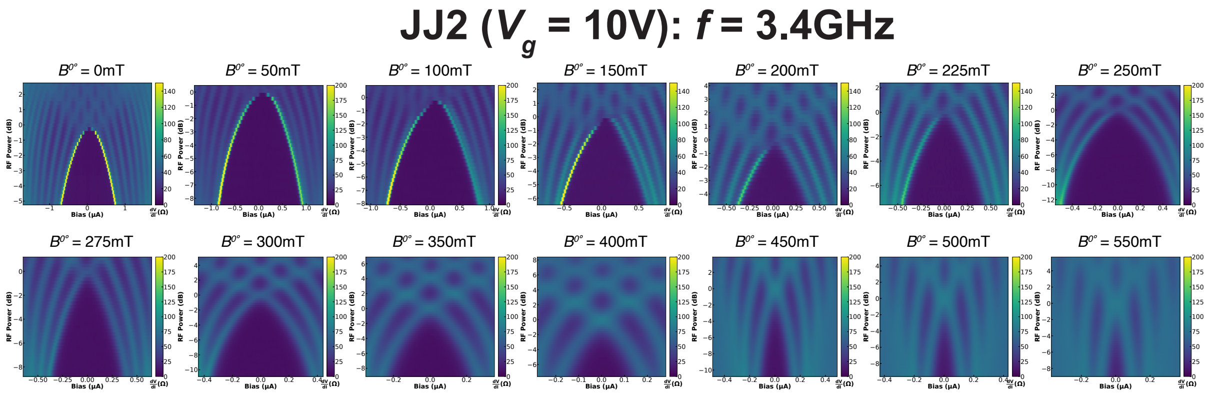

Figure **S12**: **Shapiro maps for** $f = 3.4\,\text{GHz}$ **at different values of** B^{0° **for JJ2 at** $\mathbf{V}_g = +10$**V.** $B^{0^\circ}_{co}$ is seen to be $\sim 225\,\text{mT}$.

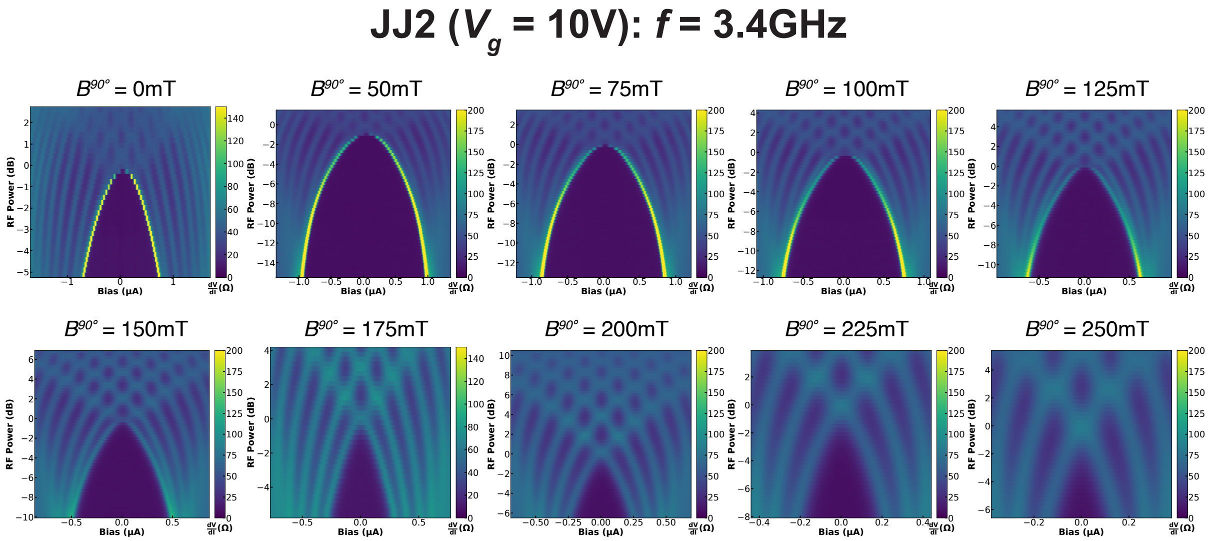

Figure **S13**: **Shapiro maps for** $f = 3.4\,\text{GHz}$ **at different values of** B^{90° **for JJ2 at** $V_g = +10\,\mathbf{V}$**.** $B^{0^\circ}_{co}$ is seen to be $\sim 200\,\text{mT}$.

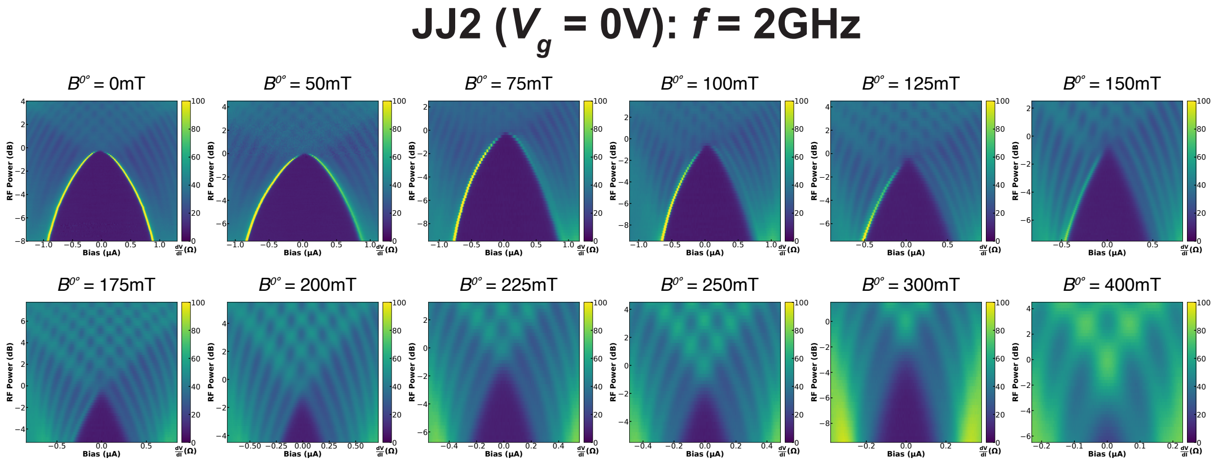

Figure **S14**: **Shapiro maps for** $f = 2\,\text{GHz}$ **at different values of** B^{0° **for JJ2 at** $V_g = 0\,\mathbf{V}$**.** $B^{0^\circ}_{co}$ is seen to be 175-200 mT.

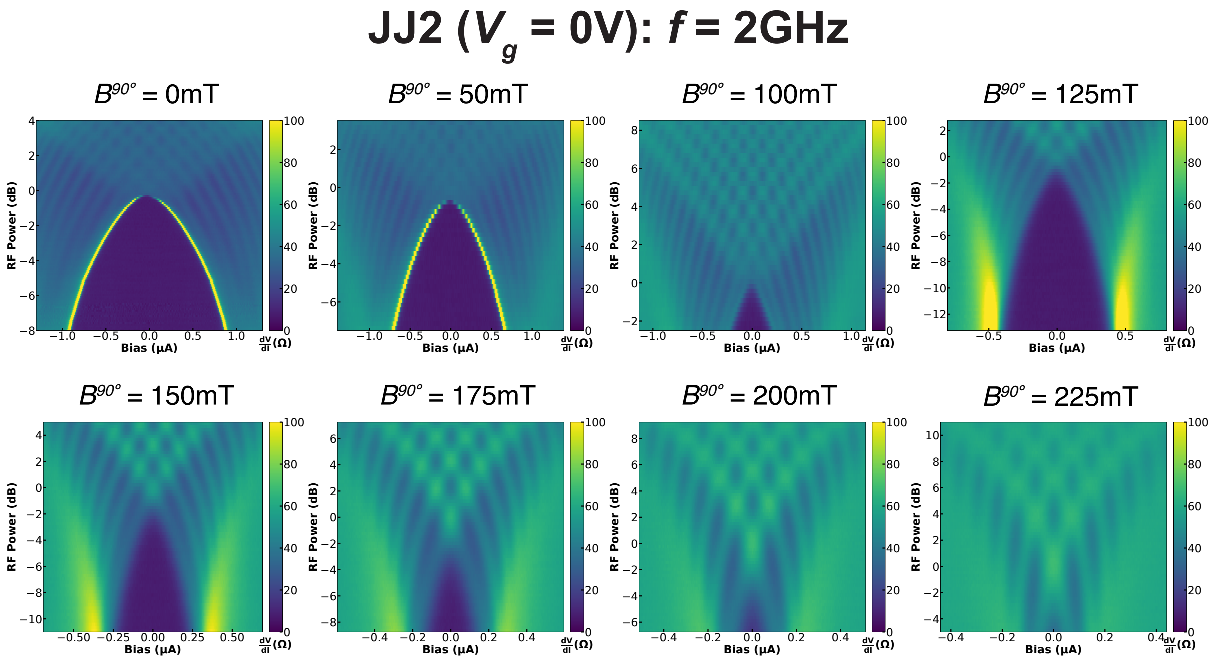

Figure **S15**: **Shapiro maps for** $f = 2\,\text{GHz}$ **at different values of** B^{90° **for JJ2 at** $V_g = 0$**V.** $B^{90^\circ}_{co}$ is seen to be $\sim 150\,\text{mT}$.

Shapiro maps. We note that even in the presence of this asymmetry, the calculated values of Q_{12} from the positive and negative sides of the current bias are quantitatively similar.

Theoretical Analysis

In this section, we qualitatively examine the Andreev bound states (ABSs) spectrum of a wide Josephson junction with an applied finite magnetic field. We discuss the primary features that allow for a robust (topologically trivial) 4π-periodic supercurrent to arise and describe the mechanisms by which a magnetic field can suppress the 4π-periodic supercurrent, causing the reappearance of odd Shapiro steps.

Tight Binding Model

To characterize the ABS spectrum, we use an effective tight-binding model that we implement using the Kwant package .[5] Consider a square lattice in the x-y plane with dimensions $(2L_{sc} + 2L_n + 1) \times (2w + 1)$ and lattice constant a. Let Ω_n, Ω_{sL}, and Ω_{sR} be the set of lattice sites in the normal, left superconducting, and right superconducting regions, respectively; let Ω be the set of all lattice sites in the system. The tight-binding version of the Hamiltonian we use is

$$H_{tb} = H_0 + H_{soc} + H_Z + H_{sc} \tag{1}$$

$$H_0 = \sum_{i\in\Omega} \psi_i^\dagger \left(4t - \mu\right) \tau_z\sigma_0\psi_i + \sum_{\langle ij\rangle\in\Omega} \psi_i^\dagger(-t)\tau_z\sigma_0\psi_j + h.c. \tag{2}$$

$$\begin{aligned} H_{soc} = &\frac{i\ell_{soc,n}}{2} \sum_{\langle ij\rangle\in\Omega_n} \psi_i^\dagger \left(\delta_{y_i,y_j}\tau_z\sigma_y - \delta_{x_i,x_j}\tau_0\sigma_x\right)\psi_j \\ &+ \frac{i\ell_{soc,sc}}{2} \sum_{\alpha=L,R} \sum_{\langle ij\rangle\in\Omega_{s\alpha}} \psi_i^\dagger \left(\delta_{y_i,y_j}\tau_z\sigma_y - \delta_{x_i,x_j}\tau_0\sigma_x\right)\psi_j + h.c. \end{aligned} \tag{3}$$

$$H_Z = \sum_{i\in\Omega_n} \psi_i^\dagger \frac{\Delta_{Z,n}}{2} \left(\sin(\theta)\tau_z\sigma_x + \cos(\theta)\tau_0\sigma_y\right)\psi_i + \sum_{\alpha=L,R}\sum_{i\in\Omega_{s\alpha}} \psi_i^\dagger \frac{\Delta_{Z,sc}}{2} \left(\sin(\theta)\tau_z\sigma_x + \cos(\theta)\tau_0\sigma_y\right)\psi_i \tag{4}$$

$$H_{sc} = -\Delta e^{i\phi/2} \sum_{i\in\Omega_{sL}} \psi_i^\dagger\tau_y\sigma_y\psi_i - \Delta e^{-i\phi/2} \sum_{i\in\Omega_{sR}} \psi_i^\dagger\tau_y\sigma_y\psi_i \tag{5}$$

where $\psi_i = \left(c_{i,\uparrow},\ c_{i,\downarrow},\ c_{i,\uparrow}^\dagger,\ c_{i,\downarrow}^\dagger\right)^T$, $c_{i,s}^\dagger$ $(c_{i,s})$ is the creation (annihilation) operator for an electron at site i with coordinate (x_i, y_i) and spin s, τ_i and σ_i are Pauli matrices in particle-hole and spin space, respectively. Here, μ is the chemical potential, $t = \hbar^2/(2m^*a^2)$, with m^* as the effective mass, is the hopping parameter (assumed to be uniform throughout the system), $\ell_{soc,n}$ is the strength of SOC coupling in the normal region, $\ell_{soc,sc}$ is the strength of SOC coupling in the superconducting region, $\Delta_{Z,n}$ ($\Delta_{Z,sc}$) are the Zeeman splitting in the normal region (superconducting region), Δ is the superconducting gap, and ϕ is the phase across the junction. For all the results presented in the main text and the following, we assume $\ell_{soc,sc} = \Delta_{Z,sc} = 0$. We have verified that qualitatively the results do not change if we set $\ell_{soc,sc}$ and $\Delta_{Z,sc}$ equal to a finite value less or equal to the ones used for the normal region. The parameters used in the calculations are given in Table 1.

Long junction modes and detachment gap

Consider a Josephson junction in the x-y plane where the weak link is in the region $-L_x/2 < x < L_x/2$ and $-w/2 < y < w/2$, and the current in the junction flows in the x-direction. Let the superconducting coherence length associated with a subband of index n_y

Table 1: Parameters used in simulations.

Δ	t	a	$\ell_{soc,n}$	L_n	L_{sc}	w	n
300 μeV	59 meV	4 nm	7.5 meV·nm	100 nm	1 μm	500 nm	4×10^{11}cm^{-2}

be $\xi_{n_y} = \hbar v_F^{(n_y)}/\pi\Delta$ where,[6]

$$v_F^{(n_y)} = \frac{2at}{\hbar}\left(\frac{\mu}{t} - \pi^2\frac{a^2}{w^2}n_y^2\right)^{1/2}. \qquad (6)$$

We see that for some n_y modes $v_F^{(n_y)}$ can be sufficiently small so that $L_x > \xi_{n_y}$. We refer to these modes as "long junction" modes.[6] The ABSs corresponding to these modes are "detached" from the continuum of states associated with the superconducting leads of the junction. We denote this detachment gap from the continuum by δ. The number of long junction modes and their detachments gaps is generally sensitive to the details of the system, e.g. doping n and width w.

When the junction has high transparency, the gap at $\phi = \pi$ is sufficiently small to allow Landau-Zener transitions when the system is diabatically driven.[6–8] At the same time, the relatively large value of δ strongly suppresses Landau-Zener transition to the continuum. The presence of ABSs with a large detachment gap and a small gap at $\phi = \pi$ leads to the presence of a 4π-periodic supercurrent, $I_{4\pi}$, when the JJ is biased. This results in a topologically trivial junction to have both 2π- and 4π-periodic supercurrent channels when driven diabatically.[9,10] We expect that the suppression by the magnetic field of the 4π-periodic supercurrent due to the long junction (non-topological) modes is responsible for the reappearance of the odds Shapiro steps for $B^\theta > B_{co}$.

Effect of magnetic field

The presence of a magnetic field suppresses the superconducting gap Δ and spin-splits the ABSs' energy level. Both of these effects contribute to a reduction of the detachment gap δ of the long junction modes responsible for the 4π behavior. When $\delta \to 0$ Andreev midgap states which undergo a Landau-Zener transition from a ground to an excited state at $\phi(\text{mod}2\pi) = \pi$ will be lost to the continuum at $\phi(\text{mod}2\pi) = 0$ before completing a 4π-periodic cycle, restoring the supercurrent to a 2π-periodicity.[11]

So far, we have focused our attention on the effect of the magnetic field on the detachment gap. The experimental results, however, show that the reappearance of the odd-Shapiro steps cannot be explained simply by tracking the evolution of δ with B^θ. To illustrate this point more clearly, for $I_{4\pi}$ we can assume:

$$I_{4\pi}(B^\theta) = I_c(B^\theta)P_\pi(B^\theta)\left(1 - P_{2\pi}(B^\theta)\right), \quad (7)$$

where I_c is the critical current of the junction, and P_ϕ is the effective probability of a Landau-Zener transition at phase ϕ. For isolated ABS states, we can write

$$P_\phi(B^\theta) = \exp\left(-\pi\frac{\Gamma(B^\theta,\phi)}{2e|V_\phi|}\right), \qquad (8)$$

where Γ is the energy gap at ϕ between the ABSs involved in the Landau-Zener transition, and $|V_\phi|$ is the instantaneous voltage across the junction when the phase is equal to ϕ. For $\phi = \pi$, $\Gamma = 2\Delta(B^\theta)(1-\tau)$,[12] where τ is the junction's transparency. For $\phi = 2\pi$, $\Gamma \approx \delta(B^\theta)$. The dependence of $I_{4\pi}$ on B^θ arises from the dependence of I_c and the energy gaps Γ at $\phi = \pi$ and $\phi = 2\pi$. Using Eqs. (7), (8) we can write:

$$\frac{I_{4\pi}(B^\theta)}{I_c(B^\theta)} = \left[\exp\left(-\pi\frac{2\Delta(B^\theta)(1-\tau)}{2e|V_\pi|}\right)\right] \left[1 - \exp\left(-\pi\frac{\delta(B^\theta)}{2e|V_{2\pi}|}\right)\right]. \qquad (9)$$

From the experimental estimates of B_{co}, we can conclude that in general the ratio $\frac{I_{4\pi}(B^\theta)}{I_c(B^\theta)}$ can grow with B^θ. On the other hand, theoretically we have that δ is linearly suppressed with B^θ, and that, in first approximation $\Delta(B^\theta) = \Delta(B^\theta = 0)[1 - B^\theta/B_c]^{1/2}$. These scalings imply that the ratio $\frac{I_{4\pi}(B^\theta)}{I_c(B^\theta)}$ should always decrease with B^θ, a result at odds with

the experimental observations.

Multi-level Landau-Zener transitions

This simple analysis shows that the naive treatment based on the assumptions of well-isolated long junction modes is not adequate. This is not surprising if we consider that for the JJs studied, due to their large width, the ABSs are not well separated, see Fig **S16**, and that upon applying a Zeeman field, a high-transparency long junction mode will shift in energy and cross many other mid-gap Andreev bound states, as shown in Fig **S17**. For ABSs spectra like the ones shown in Fig **S17**, we have a complex multi-level Landau-Zener (MLZ) problem. With many coupled energy levels evolving over some time interval, the MLZ problem quickly becomes computationally prohibitive.

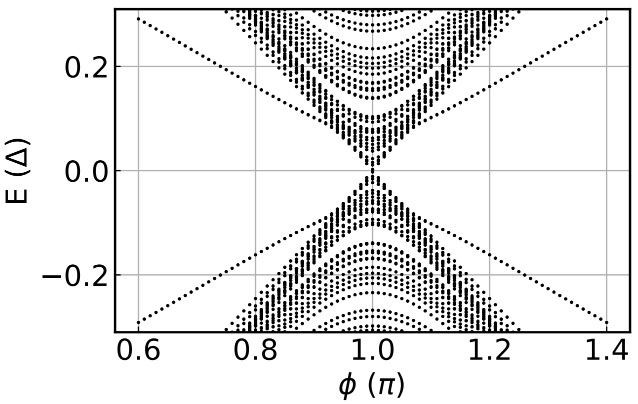

Figure **S16**: **Andreev bound state spectrum without Zeeman splitting.** JJ Andreev bound state spectrum with parameters defined in Table 1 and Zeeman splitting $\Delta_{Z,n} = 0$.

Spectra like the ones shown in Fig. **S16**, **S17** do not show a clear trend for the dependence on B^{θ} of the gaps $\Gamma(\phi = \pi)$: the gaps at $\phi = \pi$ between occupied and unoccupied ABSs appear always to be small for high-transparency, wide, JJs like the ones studied. This suggests that the dependence on B^{θ} of the effective P_{π} might be attributed to the evolution of the overlaps between the Andreev bound states involved in Landau-Zener processes. To qualitatively evaluate the multi-mode coupling in the junction,

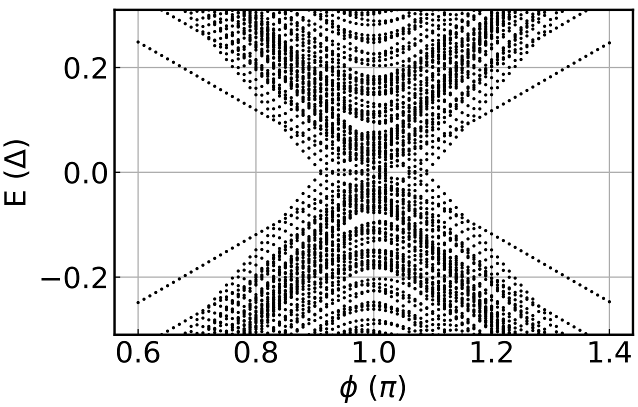

Figure **S17**: **Andreev bound state spectrum with Zeeman splitting.** JJ Andreev bound state spectrum with parameters defined in Table 1 and Zeeman splitting $\Delta_{Z,n} = 0.3\Delta$ in the 0° direction.

we calculate the overlap:

$$\mathcal{O}_{mn} = |\langle\psi_m(\phi_f)|\psi_n(\phi_i)\rangle|^2 \quad (10)$$

for $0 < \phi_i < \pi$ and $\pi < \phi_f < 2\pi$ and where $|\psi_n\rangle$ is the Andreev bound state with eigenenergy E_n. To determine whether long junction modes couple to short junction modes, we pick E_n corresponding to an occupied long junction mode and calculate $\mathcal{O}_{mn}$ for all $0 < E_m < \Delta$. The collection of overlaps form a distribution of values between 0 and 1. In the absence of a Zeeman field, the occupied long junction mode has a large overlap with only one unoccupied mode with large δ, and a very small overlap with all the other modes. A bi-modal distribution of overlaps like this will have a large kurtosis. A large kurtosis indicates that occupied long junction modes have a high probability of transitioning, for $\phi \approx \pi$, to unoccupied long junction modes and so contribute to $I_{4\pi}$. A small kurtosis indicates that occupied long junction modes are equally likely to transition to most of the low-energy, unoccupied modes present at $\phi \approx \pi$, and therefore have a large likelihood of tunneling to one of the several "short junction" modes, modes with small δ, and not contribute to $I_{4\pi}$.

We find that the kurtosis in part, depends on the values of ϕ_i and ϕ_f chosen. Despite such dependence, the kurtosis can be used to obtain qualitatively how an in-plane magnetic

field affects $I_{4\pi}$ and, therefore, the reappearance of odd-Shapiro steps. For instance, the dependence of the kurtosis on the direction θ of the magnetic field appears to explain qualitatively the dependence on θ of B_{co}/B_c measured in JJ2. To understand such dependence, we can consider the continuum model Hamiltonian, $h(k_x, k_y)$, for the normal region of the JJ:

$$h(k_x, k_y) = \frac{\hbar^2 k^2}{2m} - E_F + \lambda_{soc}\left(-k_x\sigma_y + k_y\sigma_x\right) + \frac{\Delta_Z^{(x)}}{2}\sigma_x + \frac{\Delta_Z^{(y)}}{2}\sigma_y. \quad (11)$$

For wide junctions, long junction modes have Fermi momenta mostly along the y-direction. Taking $\vec{k}_{long\ junction} \approx (0, k_y)$, we see that for the long junction modes the SOC polarizes the spins in the x-direction making the energy of these modes sensitive to the direction of the Zeeman splitting Δ_Z. Thus, in general, for wide junctions with strong SOC, the Andreev bound state spectrum will be affected differently by an in-plane magnetic field in the x- and y-direction. By calculating the ABS spectrum for different directions, and the corresponding eigenstates, we are able to obtain the dependence of the kurtosis on θ.

Fig. **S18** shows the values obtained for the kurtosis for different values of ϕ_f (keeping fixed ϕ_i) for a junction with large SOC. The blue lines show the results for the case when $B^\theta = 0$. In this case, the kurtosis is very high, suggesting that the long junction modes undergo Landau-Zener transitions that contribute to $I_{4\pi}$. The orange and green lines show the results obtained when $B^\theta \neq 0$ such that the Zeeman energy is $\Delta_Z = 0.3\Delta$ and $\theta = 90°$, orange lines, $\theta = 0°$, green lines. We see that $B^\theta \neq 0$ suppresses the kurtosis indicating an increase of the mixing of long junction modes and short-junction modes, resulting in a suppression of $I_{4\pi}$. In agreement with the experiment, specifically the data shown in Fig. 4, the results of Fig. **S18** show that the kurtosis is larger when $\theta = 90°$ than $\theta = 0°$, suggesting that, when the anisotropic suppression of I_c with B is taken into account, an in-plane field along the $\theta = 0°$ direction causes a larger suppression of $I_{4\pi}$, than an in-plane field along the $\theta = 90°$, the direction of the current.

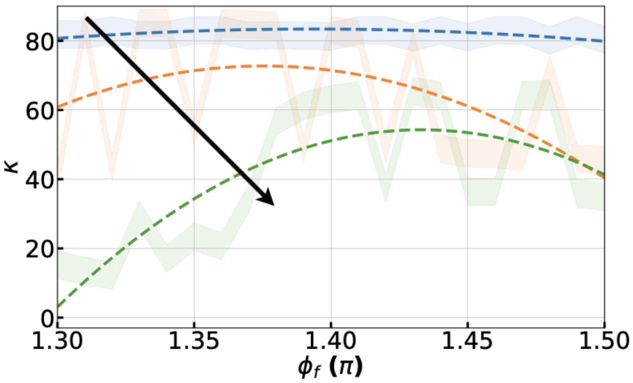

Figure **S18**: **Kurtosis versus ϕ_f for $\phi_i = 0.6\pi$.** Kurtosis of the wavefunction overlap distribution as a function of final state ϕ_f, where thick faded lines show the calculated kurtosis and dashed curves are third-order polynomial fits. Blue lines: $\Delta_Z = 0$. Orange lines: $\Delta_Z = 0.3\Delta$, $\theta = 90°$. Green lines: $\Delta_Z = 0.3\Delta$, $\theta = 0°$.

References

(1) Pauka, S. J.; Witt, J. D. S.; Allen, C. N.; Harlech-Jones, B.; Jouan, A.; Gardner, G. C.; Gronin, S.; Wang, T.; Thomas, C.; Manfra, M. J.; Gukelberger, J.; Gamble, J.; Reilly, D. J.; Cassidy, M. C. Repairing the surface of InAs-based topological heterostructures. *Journal of Applied Physics* **2020**, *128*, 114301.

(2) Suominen, H. J.; Danon, J.; Kjaergaard, M.; Flensberg, K.; Shabani, J.; Palmstrøm, C. J.; Nichele, F.; Marcus, C. M. Anomalous Fraunhofer interference in epitaxial superconductor-semiconductor Josephson junctions. *Physical Review B* **2017**, *95*, 035307.

(3) Ghatak, S.; Breunig, O.; Yang, F.; Wang, Z.; Taskin, A. A.; Ando, Y. Anomalous Fraunhofer Patterns in Gated Josephson Junctions Based on the Bulk-Insulating Topological Insulator BiSbTeSe $_2$. *Nano Letters* **2018**, *18*, 5124–5131.

(4) Wiedenmann, J.; Bocquillon, E.; Deacon, R. S.; Hartinger, S.; Herrmann, O.; Klapwijk, T. M.; Maier, L.; Ames, C.; Brüne, C.; Gould, C.; Oiwa, A.; Ishibashi, K.; Tarucha, S.; Buhmann, H.; Molenkamp, L. W. 4π-periodic Josephson supercurrent in HgTe-based topological Josephson junctions. *Nature Communications* **2016**, *7*, 10303.

(5) Groth, C. W.; Wimmer, M.; Akhmerov, A. R.; Waintal, X. Kwant: A software package for quantum transport. *New Journal of Physics* **2014**,

(6) Dartiailh, M. C.; Cuozzo, J. J.; Elfeky, B. H.; Mayer, W.; Yuan, J.; Wickramasinghe, K. S.; Rossi, E.; Shabani, J. Missing Shapiro steps in topologically trivial Josephson junction on InAs quantum well. *Nature Communications* **2021**, *12*, 78.

(7) Billangeon, P.-M.; Pierre, F.; Bouchiat, H.; Deblock, R. ac Josephson Effect and Resonant Cooper Pair Tunneling Emission of a Single Cooper Pair Transistor. *Physical Review Letters* **2007**, *98*, 216802.

(8) Domínguez, F.; Hassler, F.; Platero, G. Dynamical detection of Majorana fermions in current-biased nanowires. *Physical Review B* **2012**, *86*, 140503.

(9) Domínguez, F.; Kashuba, O.; Bocquillon, E.; Wiedenmann, J.; Deacon, R. S.; Klapwijk, T. M.; Platero, G.; Molenkamp, L. W.; Trauzettel, B.; Hankiewicz, E. M. Josephson junction dynamics in the presence of 2π - and 4π -periodic supercurrents. *Physical Review B* **2017**, *95*, 195430.

(10) Picó-Cortés, J.; Domínguez, F.; Platero, G. Signatures of a 4π -periodic supercurrent in the voltage response of capacitively shunted topological Josephson junctions. *Physical Review B* **2017**, *96*, 125438.

(11) San-Jose, P.; Prada, E.; Aguado, R. ac Josephson Effect in Finite-Length Nanowire Junctions with Majorana Modes. *Physical Review Letters* **2012**, *108*, 257001.

(12) Averin, D.; Bardas, A. ac Josephson effect in a single quantum channel. *Physical Review Letters* **1995**,
